# Evaluation of the clinical evolution and transmission of SARS-CoV-2 infection in cats by simulating natural routes of infection

**DOI:** 10.1007/s11259-022-09908-5

**Published:** 2022-03-03

**Authors:** Sandra Barroso-Arévalo, Lidia Sánchez-Morales, Jose A. Barasona, Belén Rivera, Rocío Sánchez, María A. Risalde, Irene Agulló-Ros, José M. Sánchez-Vizcaíno

**Affiliations:** 1 VISAVET Health Surveillance Center, Complutense University of Madrid, Madrid, Spain; 2 Department of Animal Health, Faculty of Veterinary, Complutense University of Madrid, Madrid, Spain; 3 Research Group in Animal Health and Zoonoses (GISAZ), Department of Anatomy and Comparative Pathology, Faculty of Veterinary, University of Cordoba, Andalusia, Spain; 4 Infectious Diseases Unit, Clinical Virology and Zoonosis Group, Maimónides Biomedical Research Institute of Cordoba (IMIBIC), Reina Sofía University Hospital, Andalusia, Spain

**Keywords:** SARS-CoV-2, Cats, Transmission, Air renovation, Routes of infection

## Abstract

Severe acute respiratory syndrome coronavirus 2 (SARS-CoV-2) is the causative agent of the current pandemic disease denominated as Coronavirus Disease 2019 (COVID-19). Several studies suggest that the original source of this virus was a spillover from an animal reservoir and its subsequent adaptation to humans. Of all the different animals affected, cats are one of the most susceptible species. Moreover, several cases of natural infection in domestic and stray cats have been reported in the last few months. Although experimental infection assays have demonstrated that cats are successfully infected and can transmit the virus to other cats by aerosol, the conditions used for these experiments have not been specified in terms of ventilation. We have, therefore, evaluated the susceptibility of cats using routes of infection similar to those expected under natural conditions (exposure to a sneeze, cough, or contaminated environment) by aerosol and oral infection. We have also evaluated the transmission capacity among infected and naïve cats using different air exchange levels. Despite being infected using natural routes and shed virus for a long period, the cats did not transmit the virus to contact cats when air renovation features were employed. The infected animals also developed gross and histological lesions in several organs. These outcomes confirm that cats are at risk of infection when exposed to infected people, but do not transmit the virus to other cats with high rates of air renovation.

**Supplementary Information:**

The online version contains supplementary material available at 10.1007/s11259-022-09908-5.

## Introduction

Attention has been paid to domestic cats (*Felis domesticus*) since the beginning of the COVID-19 (COronaVIrus Disease 2019) pandemic owing to their susceptibility to the SARS-CoV-2 pathogen (Bosco-Lauth et al. 2020; Hobbs and Reid 2020). Although the number of cats diagnosed as positive is still low when compared to the high infectivity rate among the human population, they could play an active role as viral reservoirs in the pandemic. Several studies have demonstrated that cats can be experimentally infected and are even able to transmit the virus to other cats via direct and indirect contact (Bosco-Lauth et al. 2020; Chiba et al. 2021; Hobbs and Reid 2020). Other studies have also described natural infection and antibody detection in cats exposed to infected humans or contaminated environments (Barroso-Arévalo et al. 2021; Patterson et al. 2020; Ruiz-Arrondo et al. 2020; Zhao et al. 2021), being the first detection of infected cats in France and Croatia in the first half of 2020 (Sailleau et al. 2020; Stevanovic et al. 2021). This susceptibility may be related to the high homology found between the human and cat as regards angiotensin converting enzyme 2 (ACE2) (Luan et al. 2020). This enzyme, which has a high affinity for the receptor-binding domain (RBD) of the Spike protein of SARS-CoV-2, is responsible for the virus entering the cell. These facts, together with the growing number of cats that are kept as pets, cat colonies in urban scenarios and the abundance of feral cats throughout the world, make it imperative to examine the role of this species in the current COVID-19 pandemic. Not only cats, but also large wild felines such as tigers and lions have also been naturally infected by SARS-CoV-2 (McAloose et al. 2020), including the delta variant in Asiatic lions (Mishra et al. 2021).

Experimental infection studies have demonstrated not only effective transmission between infected and contact cats (Shi et al. 2020), but also the existence of protective immunity against re-infection with SARS-CoV-2 (Chiba et al. 2021). In the aforementioned studies, naïve cats were exposed to inoculated cats by means of cohousing. Effective shedding was demonstrated through the detection of viral RNA in feces, oral swabs, nasal flush, or after-necropsy tissues. A recent study has reported that the serial passaging of the virus between cats dramatically attenuates viral transmissibility (Chiba et al. 2021), which supports the idea that cat-to-human transmission is unlikely. In this respect, most studies suggest that SARS-CoV-2 infection in cats continues to be subclinical (Bosco-Lauth et al. 2020; Cleary et al. 2020), with the exception of young animals (less than 100 days old), in which even mortality has been described (Shi et al. 2020). Despite the fact that the cats studied did not, in most cases, show any clinical signs after infection, gross and histological lesions were found in the infected animals (Chiba et al. 2021), which raises the question of whether SARS-CoV-2 infection in cats might have a greater scope. However, the above-mentioned studies did not assess natural infection conditions. For example, high doses (around 10^5^ pfu/mL) were administered using intranasal and intratracheal inoculation, which greatly differs from reality (Bosco-Lauth et al. 2020; Hobbs and Reid 2020; Shi et al. 2020). In natural conditions, domestic cats living with SARS-CoV-2 infected people are exposed to the virus by sneezes or coughs, along with contaminated surfaces. The viral loads present in these circumstances are lower than those employed in experimental assays owing to the infection route. Moreover, all of these studies were performed using a cohousing-exposure model with a high density of animals, which favors viral transmission. Fortunately, domestic and stray cats coexist in larger spaces with higher rates of ventilation.

The natural infection of domestic and stray cats has been confirmed by several studies (Barroso-Arévalo et al. 2021; Patterson et al. 2020). However, how this infection occurs under natural conditions is still an open question, since this kind of animal is subjected to less exposure. In this respect, a cough or a sneeze would appear to be the most likely pathway for a cat to become infected under natural conditions, in addition to licking contaminated surfaces (which may include their own hair). The objective of this paper is, therefore, to determine the susceptibility of cats to natural routes of infection (aerosol and licking) by simulating these two pathways for viral inoculation. We additionally studied the capacity for transmission between infected and naïve cats in two different scenarios: the first with a high level of air renewal, and the second with a lower level of air renewal than the first one. This has allowed us to evaluate the influence of high air exchanges on viral transmissibility between cats.

## Material and methods

### Ethics and animal welfare

Animal care and procedures were performed by following the guidelines of good experimental practices according to the Code of Practice for Housing and Care of Animals Used in Scientific Procedures, approved by the European Economic Community in 1986 (86/609/EEC amended by the directive 2003/65/EC) and Spanish laws (RD 53/2013). The protocol was also approved by the Community of Madrid Ethics Committee (reference PROEX 251.6/20) and by the Madrid Complutense University Ethics Committee for Animal Experiments (Project License 14/2020). The approved protocol included a detailed description of the efforts made to provide environmental enrichment and to avoid the animals undergoing any unnecessary suffering, including humane endpoints and the guidelines for euthanasia.

### Animals

Four young specific-pathogen-free (SPF) male cats of between 17 and 18 weeks old were obtained from Isoquimen (Laboratory Animal Breeder of SPF and conventional beagle dogs and cats). Temperature microchips (Biothermo®, URANOVET, S.L) were implanted in the cervical region of all the animals. These animals spent two weeks in a large room (17m^2^) located in a Biosafety Level 2 (BSL2) area at the Health Surveillance Centre (VISAVET) at the Madrid Complutense University for adaptation and socialization purposes. They were given water and dry food ad libitum and wet food was added two or three times per week. Once they were socialized, they were taken to the BSL3 area.

### Isolator and suits

**Fig. 1:**
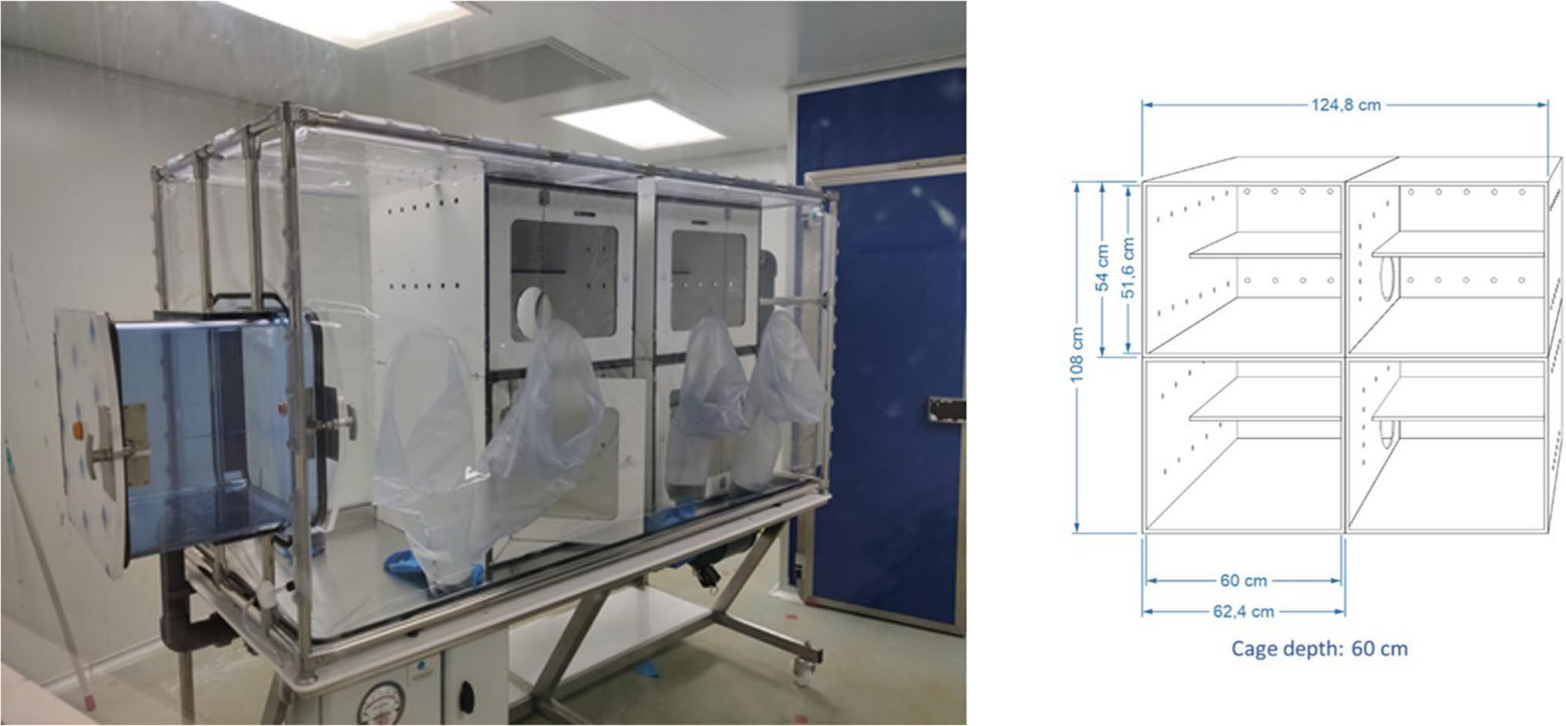
BioFlex® B90 Flexible Film Trolley Isolator with HEPA filters containing pairs inside cages measuring 124.8 × 51.6 × 60 cm used in this experiment

In the BSL3 area, the animals were located in pairs inside cages measuring 124.8 × 51.6 × 60 cm in an isolator (BioFlex® B90 Flexible Film Trolley Isolator, Livingston, UK) with HEPA filters that renovate air depending on the pressure chosen (Fig. 1). This had the same negative pressure as the box and the BSL3 area in which the experiment took place. The more negative the fixed pressure is, the higher the rate of air renovation. Two air exchange scenarios were used to study transmission: in the first scenario (high air renovation), the isolator was fixed at -50 Pascals (Pa), with 45 renovations per hour, while in the second scenario (medium air renovation), the isolator was fixed at -25 Pa, with 22.5 renovations per hour. The suits used to enter the box and sample the animals were SubiTUS® (TB-Safety AG, Aargau, Switzerland), designed for handling BSL3 pathogens.

### Virus and cells

SARS-CoV-2 MAD6 isolated from a 69-year-old male patient from Madrid (Spain) was kindly provided by Dr. Luis Enjuanes from the National Biotechnology Centre (CNB) at the Higher Council for Scientific Research (CSIC). This strain belongs to the B.1 (Pango v.3.1.16 2021–11-04) lineage.

Vero E6 cells, provided by the Instituto Carlos III (Madrid, Spain) or ATCC®, (Manassas, Virginia), were prepared in order to reproduce stocks of SARS-CoV-2. Cells were incubated at 37 °C under 5% CO2 in Gibco Roswell Park Memorial Institute (RPMI) 1640 medium with L-glutamine (Lonza Group Ltd, Basel Switzerland) and supplemented with 100 IU/ml penicillin, and 100 μg/ml streptomycin and 10% fetal bovine serum (FBS) (Merck KGaA, Darmstadt, Germany). SARS-CoV-2 titers were determined as being the amount of virus causing cytopathic effects in 50% of infected cultures (TCID_50_/ml). Genomic stability after passages of the virus in cells was evaluated by sequencing the whole genome of the original virus and the inoculum used for this experiment as described in (Barroso-Arévalo et al. 2021).

### Infection

The animals were sedated before all the procedures using dexmedetomidine 0.01 mg/kg (Dexmopet 0.5 mg, Fatro Ibérica S.L, Barcelona, Spain) and butorphanol 0,4 mg/kg (Torphadine 10 mg/ml, Fatro Ibérica S.L, Barcelona, España) (Nagore et al. 2013). The first animal (infected animal 1, INF1) was sprayed with 1 mL of 3.16 × 10^5^ TCID_50_ of SARS CoV-2 MAD6 three different times on two consecutive days, thus simulating a human cough or sneeze. This animal was euthanized on day 11 post-infection (DPI). On its 3 DPI, another cat (contact animal 1, CNT1) was cohoused with it in order to study direct contact transmission between cats with a high rate of air renovation (scenario 1) (Fig. 1). The second animal infected (infected animal 2, INF2) was inoculated with the same dose as the first one (1 mL of 3.16 × 10^5^ TCID_50_ of SARS CoV-2 MAD6), twice on two consecutive days (once per day) using another route of infection: distributing the virus all over the animal’s hair, thus simulating a licking infection model (oral). It was, like the first one, euthanized on 11 DPI. On its 4 DPI, another animal (contact animal 2, CNT2) was cohoused as a sentinel contact control (Fig. 1). The isolator was fixed at -50 Pascals (negative pressure) with 45 air renovations occurring every hour (scenario 1 = high air exchange).

**Fig. 2:**
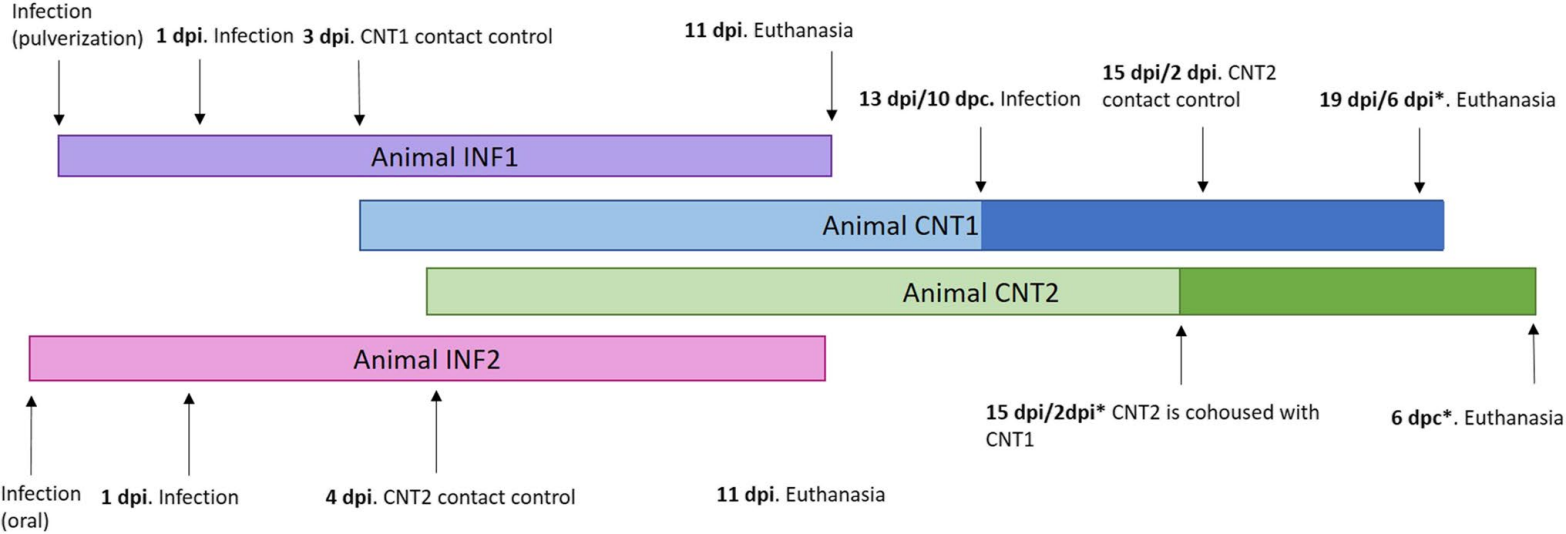
Diagram of inoculation of infected animal 1 (INF1) and sentinel contact control (contact animal 1, CNT1); inoculation of infected animal 2 (INF2) and sentinel contact control (contact animal 2, CNT2); and inoculation of contact animal 1 (CNT1) cohoused with a sentinel contact control (CNT2). DPI: day post-infection. DPC: day post-contact. *The asterisk indicates the period after which CNT1 became infected

As CNT1 did not become infected, it was subsequently inoculated on 13 DPI, which corresponded with its 10 DPC (day post-contact) with the same dose and the same inoculation route as INF1. 10 days has been shown to be enough incubation period for cats for developing SARS-CoV-2 infection after contact with infected cats (Bosco-Lauth et al. 2020; Gaudreault et al. 2020), therefore, CNT1 was considered as not infected cat since all the samples taken from this animal tested negative for SARS-CoV-2 detection. On its 2 DPI* (the asterisk indicates the period after CNT1’s infection), CNT2 (naïve animal), was introduced as a sentinel contact control (Fig. 2). The isolator pressure was reduced to -25 Pascals in order to reduce the air renovation to 22.5 renovations per hour (scenario 2 = medium air exchange). CNT1 was euthanized on its 6 DPI*, and CNT2 was euthanized on its 6 DPC*.

All the animals were euthanized intravenously using 3–5 ml of sodium pentobarbital (Dolethal, Vetoquinol Especialidades Veterinarias, S.A, Madrid, España) and were subjected to a systematic necropsy in order to assess the pathological changes in the tissue samples obtained from them.

### Animals sampling

The cats were observed on a daily basis in order to monitor clinical signs such as fever, loss of body weight, and depression, along with any respiratory and digestive symptoms. Special feeders with temperature microchip readers [Sure Petcare Feeders, Sure Petcare (SureFlap Ltd), Ground Floor, Building 2030, Cambourne Business Park, Cambourne, United Kingdom] were used. These special feeders made it possible to record each animal’s body temperature every time it ate. Blood, serum, oropharyngeal and rectal swabs, along with surface sponges rubbed over the animals’ hair for environmental RNA detection purposes, were collected every day during the experiment. All the samples were taken under sedation (dexmedetomidine 0.01 mg/kg, butorphanol 0.4 mg/kg). Blood was obtained via the venipuncture of the cephalic, jugular, or internal saphenous vein. Whole blood was collected in EDTA (Ethylenediaminetetraacetic acid) tubes, while serum samples were collected in a tube without an anticoagulant. The swabs were collected in DeltaSwab® Virus 3 ml with viral transport media (MTV) (Deltalab S.L., Cataluña, Spain). 3 M™ Dry Sponges (3 M, Minnesota, USA) were used to detect environmental RNA on the animals’ skin and hair. These sponges were pre-hydrated with 15 ml of an isotonic surfactant liquid that inactivates the virus but preserves the genetic material (Martínez-Guijosa et al. 2020). All the samples were immediately processed and analyzed in order to avoid the viral degradation of the RNA.

After systematic necropsy, samples of brain, nasal turbinates, thymus, tonsils (palatine, pharyngeal, and lingual), salivary glands (parotid and mandibular), trachea, the lobes of each lung (right and left cranial, right and left caudals, middle and accessory), heart, spleen, liver, kidney, adrenal gland, gonads, stomach, several sections of the intestine (duodenum, jejunum, ileum, ileocecal valve, colon, and rectum), and lymph nodes (submandibular, parotid, retropharyngeal, tracheobronchial, mediastinal, gastrohepatic, mesenteric and ileocecal) were fixed in 10% buffered formalin and routinely processed for histopathological studies, in addition to being introduced into 50 ml tubes with 5 ml of PBS (Phosphate-buffered saline) and homogenized for their subsequent analysis by means of PCR.

### RNA extraction and reverse transcription-quantitative PCR (RT-qPCR)

Total RNA was extracted using the column-based High Pure Viral Nucleic Acid Kit (Roche, Basel, Switzerland), according to the manufacturer’s instructions. Total RNA was suspended in RNase/DNase-free water and stored at -80ºC.

The detection of SARS-CoV-2 RNA was performed using the envelope protein (E)-encoding gene (Sarbeco) and two targets (IP2 and IP4) of the RNA-dependent RNA polymerase gene (RdRp) in an RT-qPCR protocol established by the WHO in accordance with its guidelines (https://www.who.int/emergencies/diseases/novel-coronavirus-2019/technical-guidance/laboratory-guidance) (Corman et al. 2020). The primer sets used are detailed in Table 1. RT-qPCR was carried out using the SuperScript III Platinum One-Step RT-qPCR Kit (ThermoFisher, Massachusetts, USA), according to the protocol cited above, in a CFX Connect™Real-Time PCR Detection System (BioRad, Berkeley, USA). A positive Ct (cycle threshold) cut-off of 40 cycles was used. Samples that amplificated at least 2 targets with Ct-values < 40 and confirmed by sequencing were considered positive, according to OIE guidelines (OIE 2021).

**Table 1:** Primer sequences and amplified fragment sizes in base pairs

Primer target	Sequence 5’ − 3’	PCR fragment size
Gene RdRp/ nCoV_IP2		
nCoV_IP2 − 12669Fw	ATGAGCTTAGTCCTGTTG	108 bp
nCoV_IP2 − 12759Rv	CTCCCTTTGTTGTGTTGT	
nCoV_IP2 − 12696b Probe( +)	AGATGTCTTGTGCTGCCGGTA [5']Hex [3']BHQ − 1	
Gene RdRp/ nCoV_IP4		
nCoV_IP4 − 14059Fw	GGTAACTGGTATGATTTCG	107 bp
nCoV_IP4 − 14146Rv	CTGGTCAAGGTTAATATAGG	
nCoV_IP4 − 14,084 Probe( +)	TCATACAAACCACGCCAGG [5']Fam [3']BHQ − 1	
Gene E/ E_Sarbeco		
E_Sarbeco_F1	ACAGGTACGTTAATAGTTAATAGCGT	125 bp
E_Sarbeco_R2	ATATTGCAGCAGTACGCACACA	
E_Sarbeco_P1	ACACTAGCCATCCTTACTGCGCTTCG [5']Fam [3']BHQ − 1	

### Neutralizing antibody detection

The SARS-CoV-2 surrogate virus neutralization test (GenScript, Inc., NJ, USA) was used as a screening test for neutralizing antibody detection, according to the manufacturer’s instructions.

All positive results were evaluated using a virus neutralization test (VNT). Briefly, 25 μL of two-fold serially diluted sera were incubated with 25 μL of 100 TCID_50_/ml of SARS-CoV-2 in 96-well plates at 37 °C with 5% CO_2_. At 1-h post-incubation, 200 μL of Vero E6 cell suspension were added to the virus-serum mixtures, and the plates were incubated at 37 °C with 5% CO_2_ for 3–4 days. The neutralization titers were determined at 3–4 days post-infection. The titer of a sample was recorded as the reciprocal of the highest serum dilution that provided 100% neutralization of the reference virus, which was determined by visualizing the cytopathic effect (CPE). Moreover, cell viability after VNT was determined by employing a violet crystal assay in order to confirm the results observed using microscopy. This was done as follows: at the end of the VNT, the cells were dried and fixed with ethanol 100%, and 200 µl of 0.5% crystal violet solution (Sigma-Aldrich, Missouri, USA) were added and incubated for 20 min at room temperature. Finally, the crystal violet was removed for the visualization of the plaques. Cell viability was determined by comparing each well with both the virus and the cell control wells.

### Histopathological Analysis

Tissue samples were fixed in 10% phosphate-buffered formalin for 24–72 h, and then immediately dehydrated in ethanol, immersed in xylol, and embedded in paraffin wax by employing an automatic processor. Sections of 4 µm were stained with hematoxylin and eosin and examined microscopically using a Modular Microscopy BX43 (Olympus, Shinjuku, Tokyo, Japan). The lesions were evaluated by two experienced observers and blinded as regards which animal was being analyzed.

### Statistical analyses

Data exploration, analyses, and graphs were performed using SPSS 20 (IBM, Somar, NY, USA). Differences in Ct-values and temperatures among different sampling periods were assessed for statistical significance using the non-parametric Mann–Whitney U test (MW-U test), since the data for all variables had a skewed distribution. Statistical tests were set at a significance level of 95%; i.e., *p* < 0.05. The percentage of inhibition obtained from the SARS-CoV-2 surrogate virus neutralization test was used to create a graphical representation of neutralizing antibody production, while Ct-values were used for the representation of viral loads based on RT-qPCR.

## Results

### Clinical signs

None of the cats showed any clinical signs of the disease throughout the study, with the exception of INF2 (infected by oral route), which had diarrhea on the second day post-infection. Body weights were maintained over time. The temperature was registered 30–40 times per day, depending on the cats’ activity, and there were variations in the infected animals over time (see Additional file 1, 2 and 3). In the first infected animal (INF1), the highest temperatures were found on 4–5 DPI, with the greatest value being 39.7ºC, which was significantly different from the other days (MW-U test; *U* = 13,110; *p* < 0.01). However, the highest temperatures attained by the second infected animal (INF2) occurred on 2–3 DPI, with the greatest value being 39.9ºC, which was also significantly different from the other days of the experiment (MW-U test; *U* = 8565.5; *p* < 0.01). However, no changes in temperature were seen in CNT1 and CNT2 during the period in which the experiment was carried out.

### Viral replication and neutralizing antibody production

**Fig. 3:**
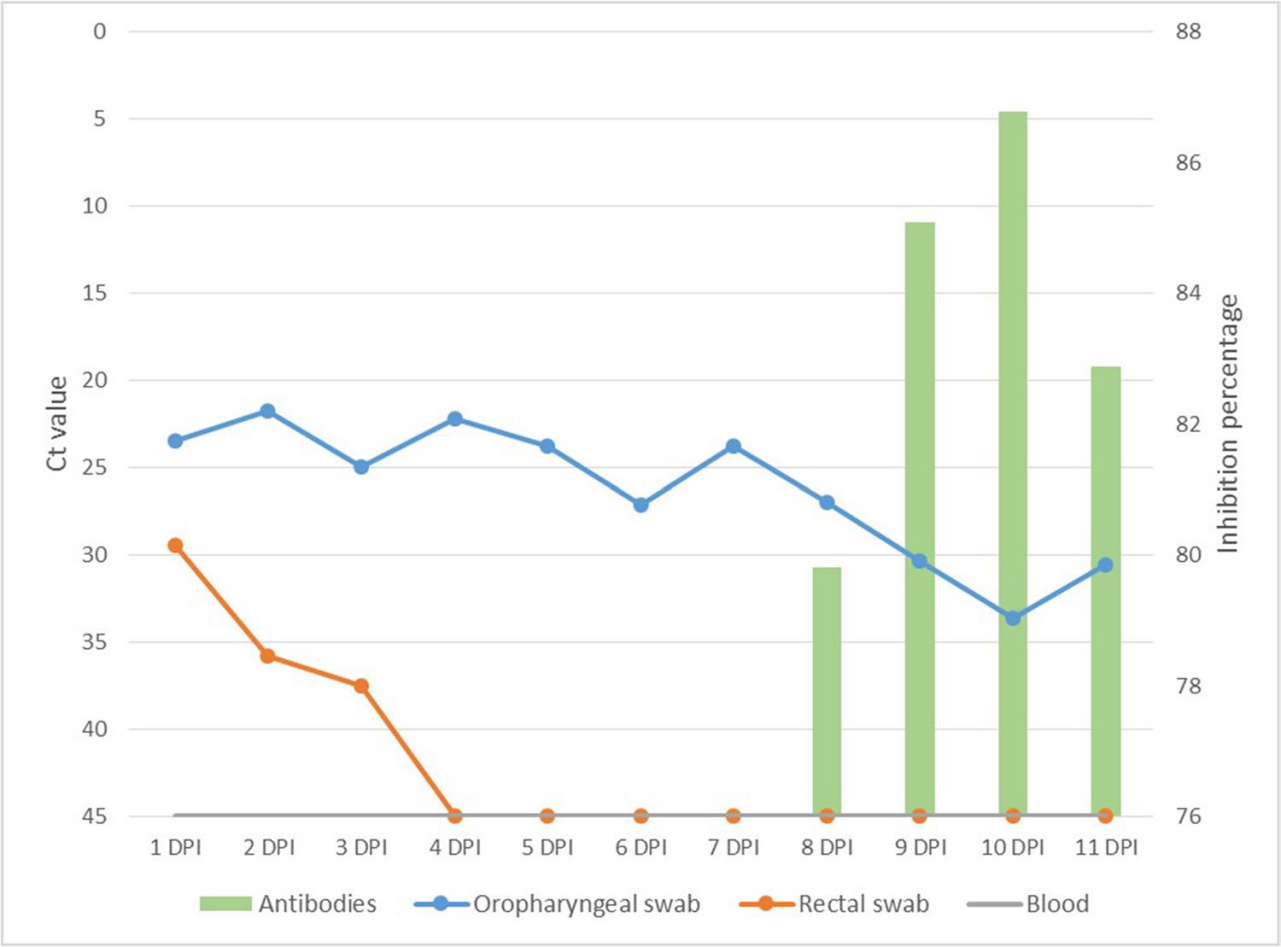
Viral loads based on Ct value measured by employing RT-qPCR on both oropharyngeal and rectal swabs as well as blood (left axis; continuous lines), and neutralizing antibody production based on the percentage of inhibition measured by employing SARS-CoV-2 surrogate virus neutralization test (right axis; bars) with infected animal 1 (IFN1)

**Fig. 4:**
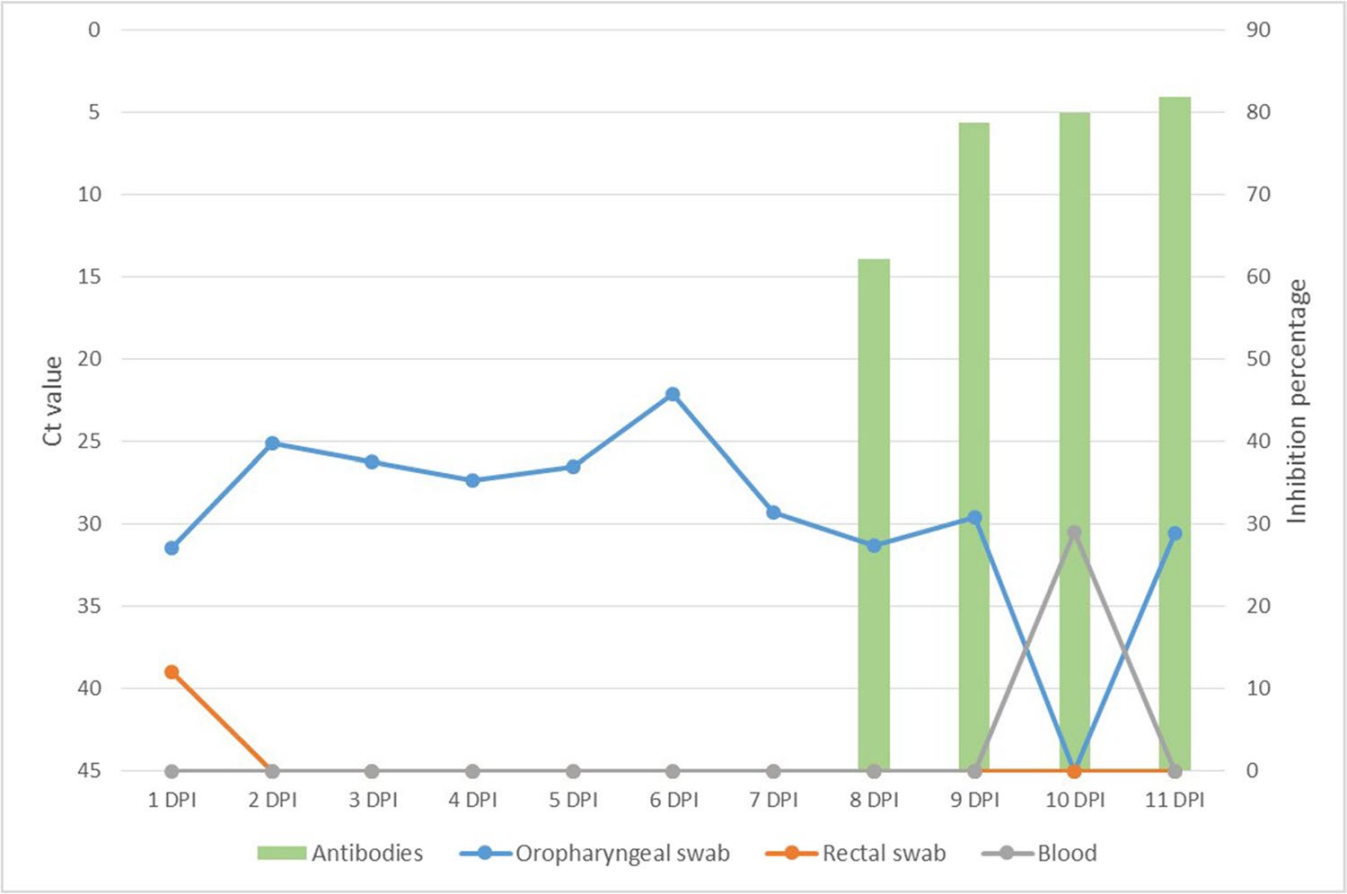
Viral loads based on Ct value measured by employing RT-qPCR on both oropharyngeal and rectal swabs as well as blood (left axis; continuous lines), and neutralizing antibody production based on percentage of inhibition measured by employing SARS-CoV-2 surrogate virus neutralization test (right axis; bars) with infected animal 2 (IFN2)

Viral replication was detected in both INF1 and INF2 from 1 DPI until the day of euthanasia as regards the oropharyngeal swabs and was also detected on the rectal swabs obtained from INF1 the first three days after inoculation. Viral loads based on Ct values were higher on the first 8 days (MW-U test; *U* = 1.00, *p* = 0.001) for both infected animals (INF1: Average Ct = 23.86, CI 95% = 0.03; INF2: Average Ct = 26.85, CI 95% = 0.04). The viral loads decreased in both infected animals from 8 DPI (INF1: Average Ct = 30.36, CI 95% = 0.05; INF2: Average Ct = 30.49, CI 95% = 0.02) (Table 2), coinciding with the starting point of neutralizing antibody production, which was measured by employing the SARS-CoV-2 surrogate virus neutralization test and VNT (Figs. 3 and 4). In the case of CNT1, values were registered only until its 6 DPI*, signifying that no neutralizing antibodies were produced (Fig. 4). CNT2 did not produce any neutralizing antibodies as it was not infected during its contact period.

**Table 2:** Mean Ct values obtained from oropharyngeal swabs in RT-qPCR from 1–7 DPI and from 8–11 DPI in INF1 and INF2. Confidence intervals (CI 95%) are represented

Animal ID	Ct value in PCR between 1–7 DPI		Ct value in PCR between 8–11 DPI	
	Average Ct value	CI 95%	Average Ct value	CI 95%
INF1	23.86	0.03	30.36	0.05
INF2	26.85	0.04	30.49	0.02

As described above, neutralizing antibody production started at 8 DPI, peaking at 10 DPI in the case of INF1 (percentage of inhibition 86.79%) and on the day of euthanasia in the case of INF2 (percentage of inhibition, 81.87%).

**Fig. 5:**
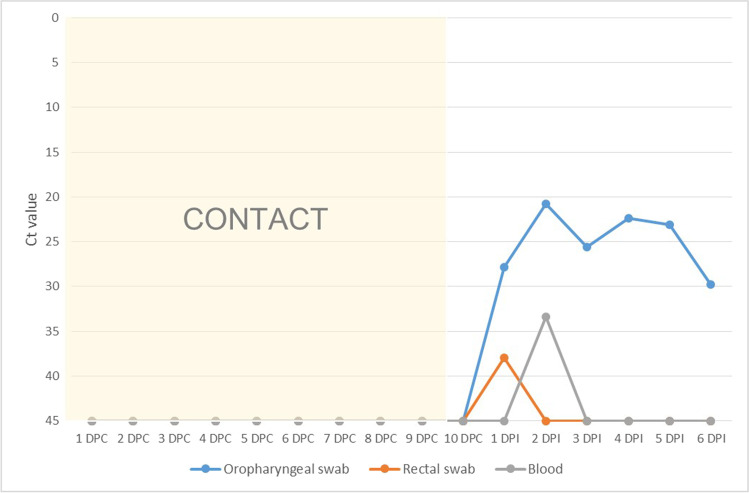
Viral loads based on Ct value measured by employing RT-qPCR on both oropharyngeal and rectal swabs as well as blood during contact and infection period in CNT1. Neutralizing antibodies were not produced before euthanasia (6 days post-inoculation)

As the RT-qPCR evaluation did not show any positive results for CNT1 during its contact period with INF1, we considered it to be a non-infected animal. On its 10 DPC, the animal was inoculated by the same pathway as INF1. CNT1 was then successfully infected, and attained positive results to RT-qPCR from day 1 post-infection until it was euthanized (Fig. 5). Interestingly, this animal also had viremia at 2 DPI (average Ct targets = 31.79. CI 95% = 0.13).

### Viral loads on sponges used on the animals’ hair (environmental contamination)

**Fig. 6:**
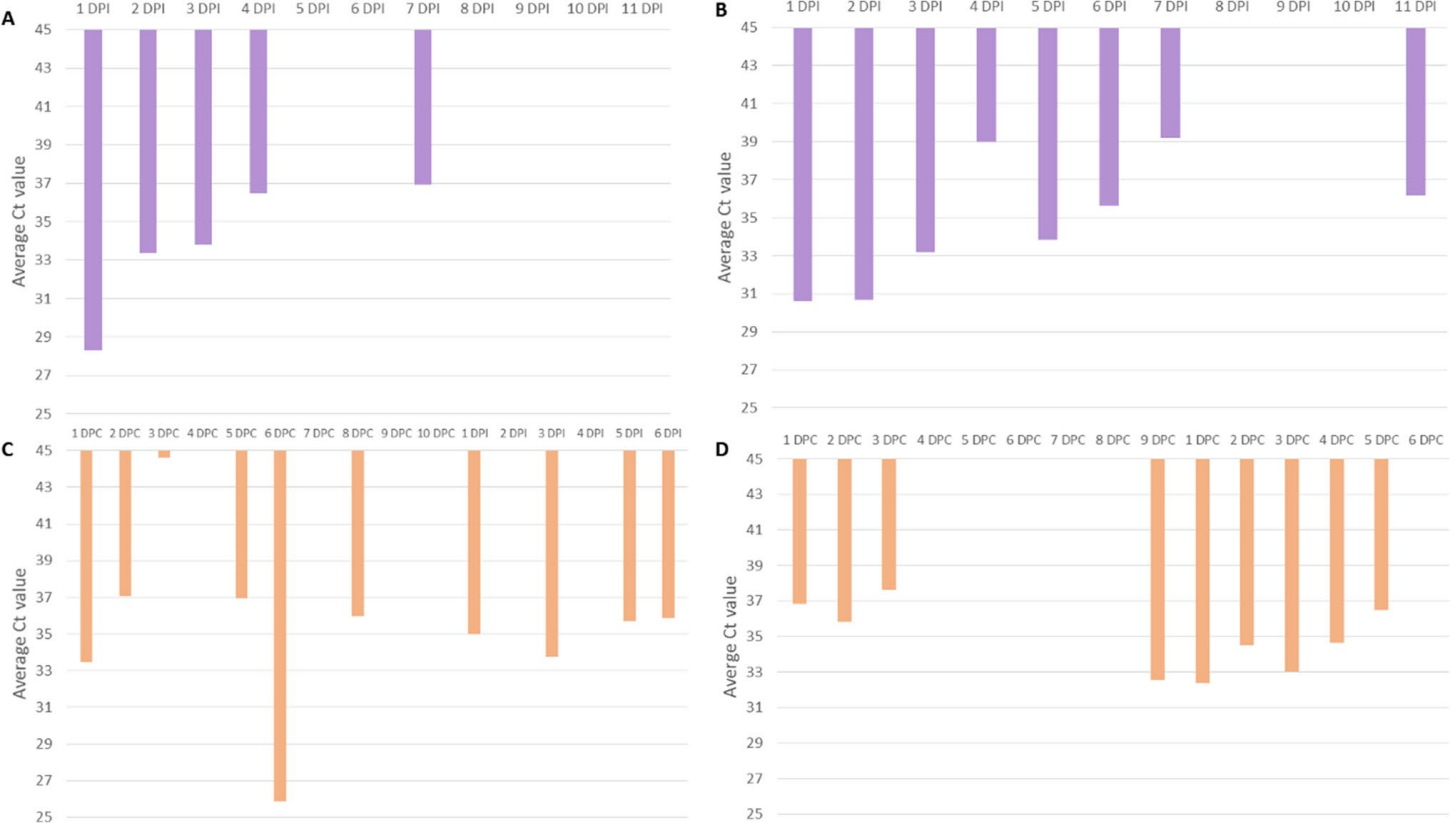
Average viral loads based on Ct values on sponges taken from INF 1 (**A**), INF2 (**B**), CNT1 (**C**), and CNT2 (**D**) during the experiment

Environmental sponges were used on the animals’ hair every day from the beginning of the experiment. Viral RNA was noticed on the sponges used on infected animals INF1 and INF2, and on the contact sentinel cats (CNT1 and CNT2), which were cohoused with the infected animals. However, the remaining samples (swabs, serum, and blood) taken were always negative for these animals during their sentinel contact period (Fig. 6).

### Transmission study

Transmission between infected and contact cats was evaluated in two different scenarios. In the first one (scenario 1), a higher air exchange was used by fixing the isolator at -50 Pascals (negative pressure) with 45 air exchanges per hour. INF1 was infected by aerosol, while INF2 was infected orally. None of the contact animals (CNT1 and CNT2) became infected with this air renovation, since both animals tested negative as regards all the samples taken during the entire period of contact.

In the second scenario (scenario 2), a lower air exchange environment was created by fixing the isolator at -25 Pascals (negative pressure) with 22.5 air exchanges occurring every hour. In this second scenario, we infected CNT1 and cohoused it with CNT2 to be its sentinel contact on 1 DPI* of the inoculated animal. Despite the fact that CNT1 was successfully infected (as described in the previous section), CNT2 did not attain any positive results to RT-qPCR for any of the samples taken during the experiment, and this was also the case of its tissues after necropsy.

### Gross lesions

**Fig. 7:**
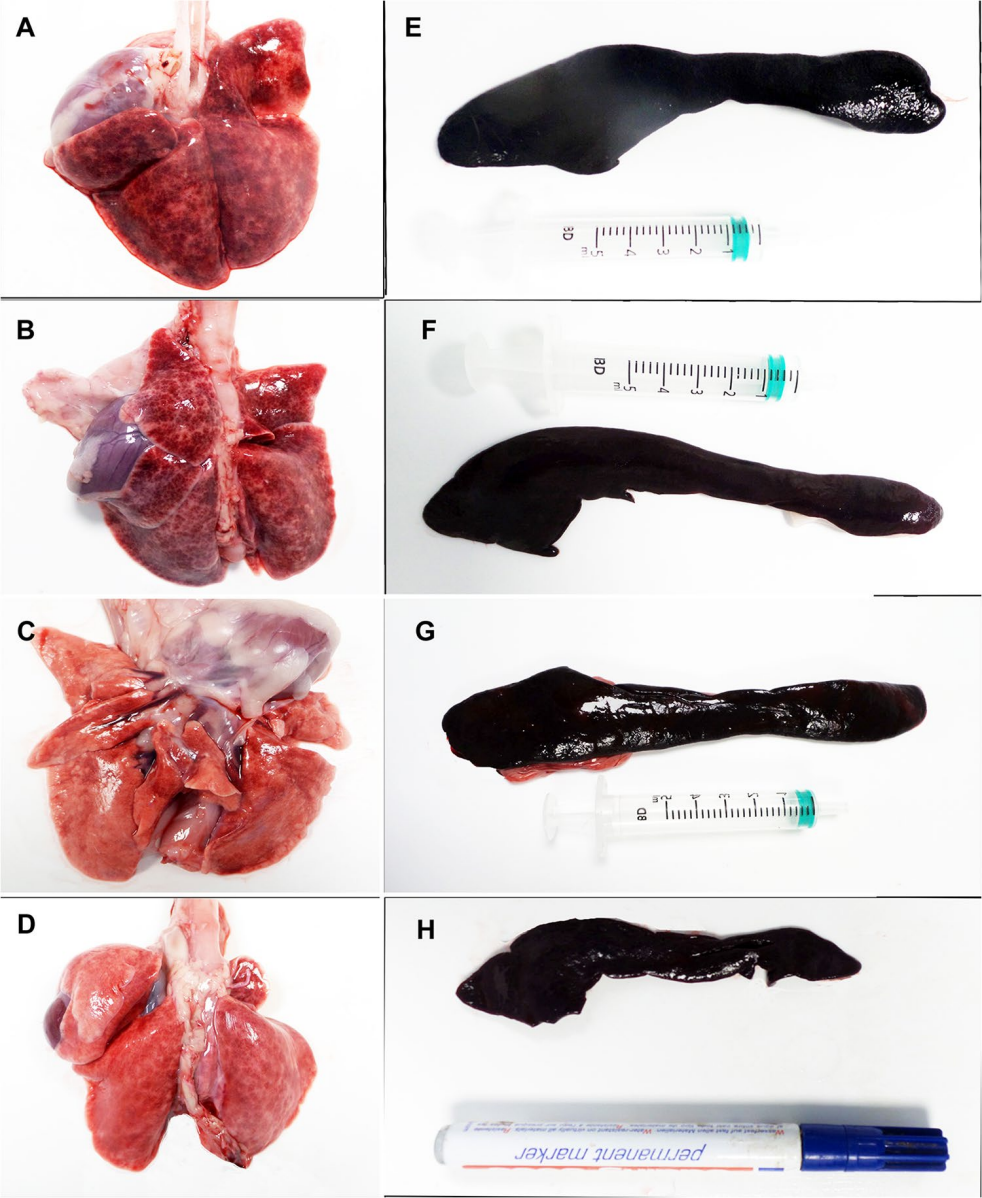
Gross lesions were found in tissues of the inoculated animals (INF1, INF2, and CNT1). Congestion and interstitial pneumonia were found in the three animals (INF1 = A; INF2 = B; CNT1 = C), and animal INF1 (**A**) had alveolar edema and seromucosal tracheitis, while animal CNT2 lungs were perfectly normal (CNT2 = D). The spleens of all three animals were enlarged and congested (INF1 = E; INF2 = F; CNT1 = G), while animal CNT2 had no gross lesions (CNT2 = H)

In general terms, gross lesions were greater in INF1 (infected by aerosol) and INF2 (Fig. 7B) (infected by oral route) than in CNT1 (infected by aerosol after its contact period). The lungs showed signs of intense congestion and there were images compatible with moderate interstitial pneumonia, with these lesions being slighter in CNT1 than in the other animals (Fig. 7C). Moreover, INF1 had alveolar edema and tracheitis (Fig. 7A). Splenic congestion and splenomegaly were detected in the three infected animals (Fig. 7E-G). The small intestine in all three infected animals exhibited mucosal thickening together with slightly larger Peyer’s patches.No gross lesions were observed in contact animal CNT2 (Fig. 7D, H).

### Histopathological evaluation

**Fig. 8:**
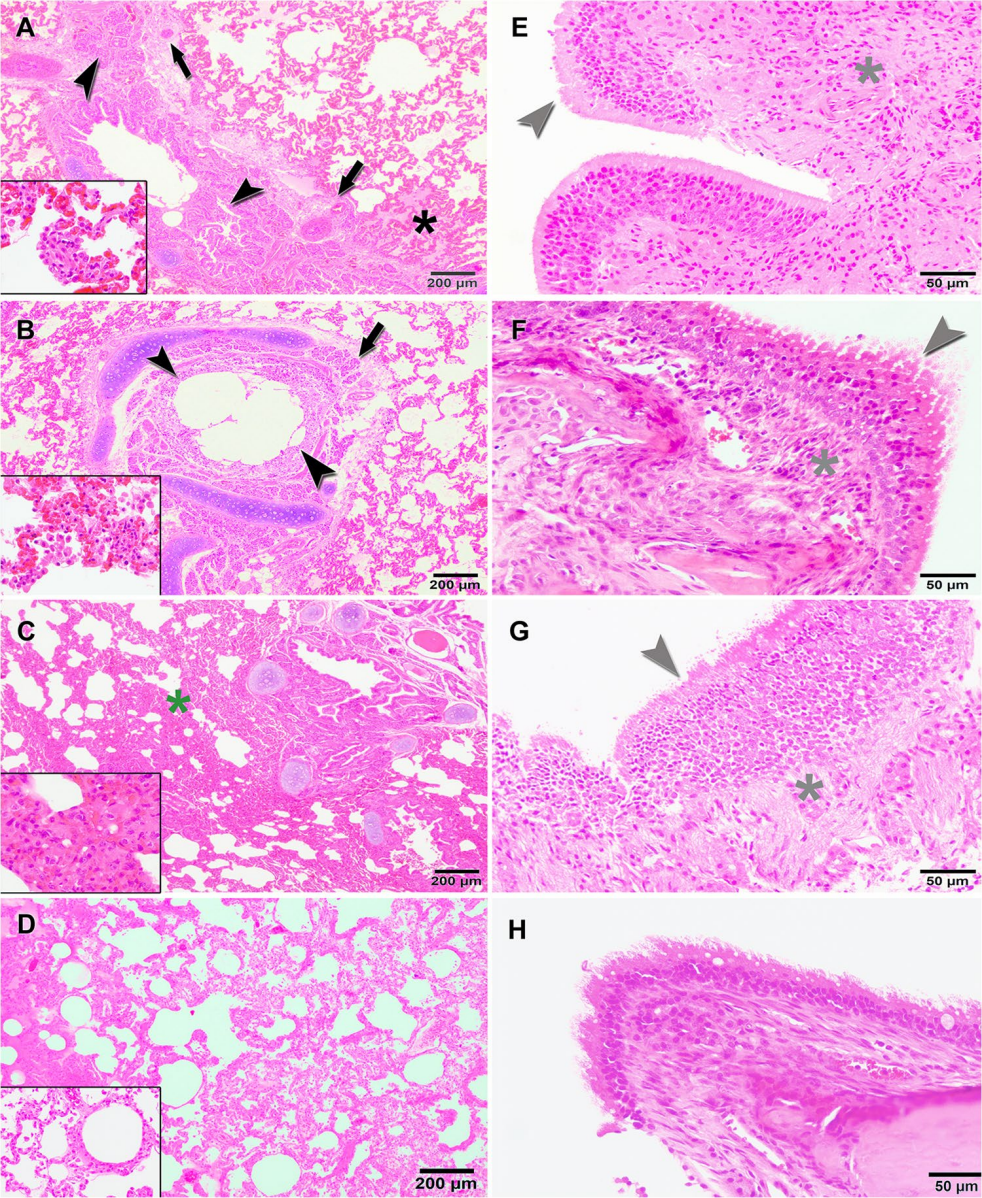
SARS-CoV-2 infection caused several respiratory lesions in the cats. INF1 (**A**) and INF2 (**B**), both euthanized at 11 DPI, had an intense congestion, alveolar (black asterisk), and perivascular edema (arrows), along with bronchial glandular and epithelial hyperplasia (arrowheads). Hyperplasia of type II pneumocytes (A, inset) and alveolar macrophages (B, inset) was also observed in these animals. These lesions were slighter in CNT1 (**C**), but there was a greater alveolar septal thickening as a result of light-to-severe interstitial aggregations (green asterisk) composed mainly of lymphocytes and macrophages (C, inset). Epithelial hyperplasia (grey arrowheads) and mononuclear infiltrates were observed in the lamina propria (grey asterisks) of the nasal turbinates of INF1 (**E**), INF2 (**F**), and CNT1 (**G**). No histological lesions were observed in tissues from CNT2 (**D**, **H**)

The histopathology confirmed pulmonary lesions compatible with interstitial pneumonia. The principal findings were vascular alterations, such as intense congestion, alveolar and perivascular edema, along with small interstitial and alveolar hemorrhages in the lung. These alterations appeared in a more severe form in INF1 and INF2 than in CNT1 and were associated with the perivascular infiltrate of lymphocytes and macrophages, along with hyperplasia of alveolar macrophages and type II pneumocytes (Fig. 8A, B). In contrast, the pulmonary parenchyma of CNT1 had a greater alveolar septal thickening as a result of light-to-severe interstitial aggregations composed mainly of lymphocytes and macrophages (Fig. 8C). The bronchial changes were similar in all three animals and were characterized by glandular and epithelial hyperplasia with INF1 exhibiting greater mucus production. Inflammatory changes were also observed microscopically in nasal mucosa, in which evidence of epithelial hyperplasia and moderate mononuclear infiltrates were found in the lamina propia, together with some neutrophils in INF2 (Fig. 8E, F and G). No lesions were observed in animal CNT2 (Fig. 8D, H).

**Fig. 9:**
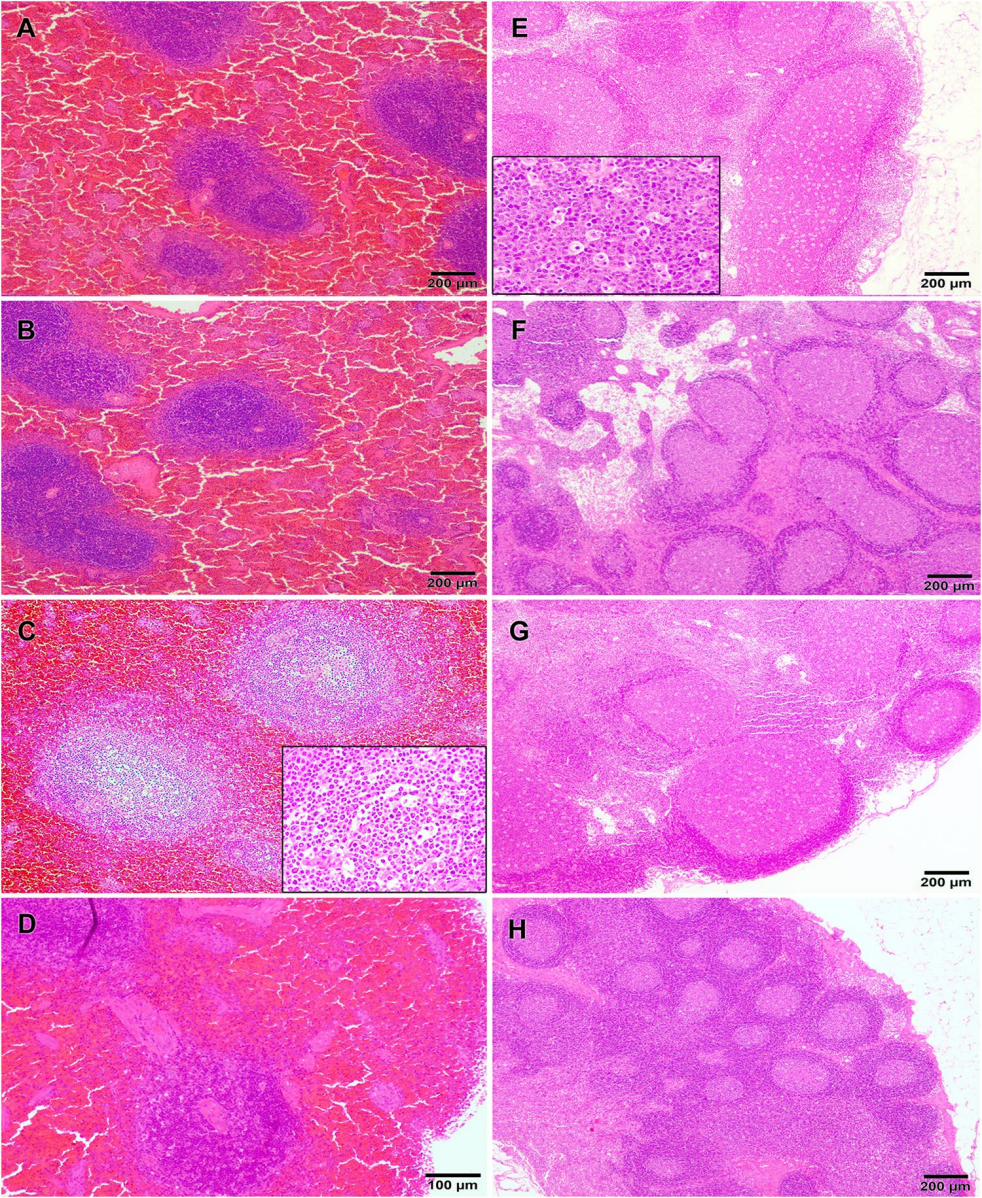
Pathological findings found in lymphoid tissues after SARS-CoV-2 infection in cats. Hyperemic splenomegaly was observed in INF1 (**A**), INF2 (**B**), and CNT1 (**C**), also accompanied by moderate lymphoid depletion in this last animal (C and C inset). A decrease in lymphocytes and images of apoptosis within germinal centers with evidence of pyknosis, cellular fragmentation, and macrophages with engulfed cell debris (tingible body macrophages) (E, inset) were also evident in several of the lymph nodes of INF1 (**E**), INF2 (**F**) and CNT1 (**G**). No histological lesions were observed in tissues from CNT2 (**D**, **H**)

Microscopic examination also confirmed splenic congestion, characterized by dilated splenic sinuses with a large number of red blood cells and widely separated germinal centers. The lesions were similar in the three infected animals, and a mild lymphoid depletion was additionally observed in CNT1 (Fig. 9A, B, and C). In this respect, a severe multifocal lymphoid depletion was also evident in several lymph nodes, which was characterized by a decrease in lymphocytes and presence of apoptosis within germinal centers, with evidence of pyknosis, cellular fragmentation, and macrophages with engulfed cell debris (tingible body macrophages) (Fig. 9E, F and G). These findings were observed in the three infected animals and were also detected in the pharyngeal tonsil of INF1, the Peyer’s patches of INF2, and the thymus of CNT1. Moreover, vascular alterations such as hyperemia and petechial hemorrhages were evident in some of the lymph nodes and tonsils of INF1 and INF2, and in the thymus of CNT1. No histological lesions were observed in the microscopical examination of CNT 2 tissues (Fig. 9D, H).

Histopathological lesions were not present in the other organs in any of the infected animals analyzed.

### Viral replication in infected animal tissues

**Fig. 10:**
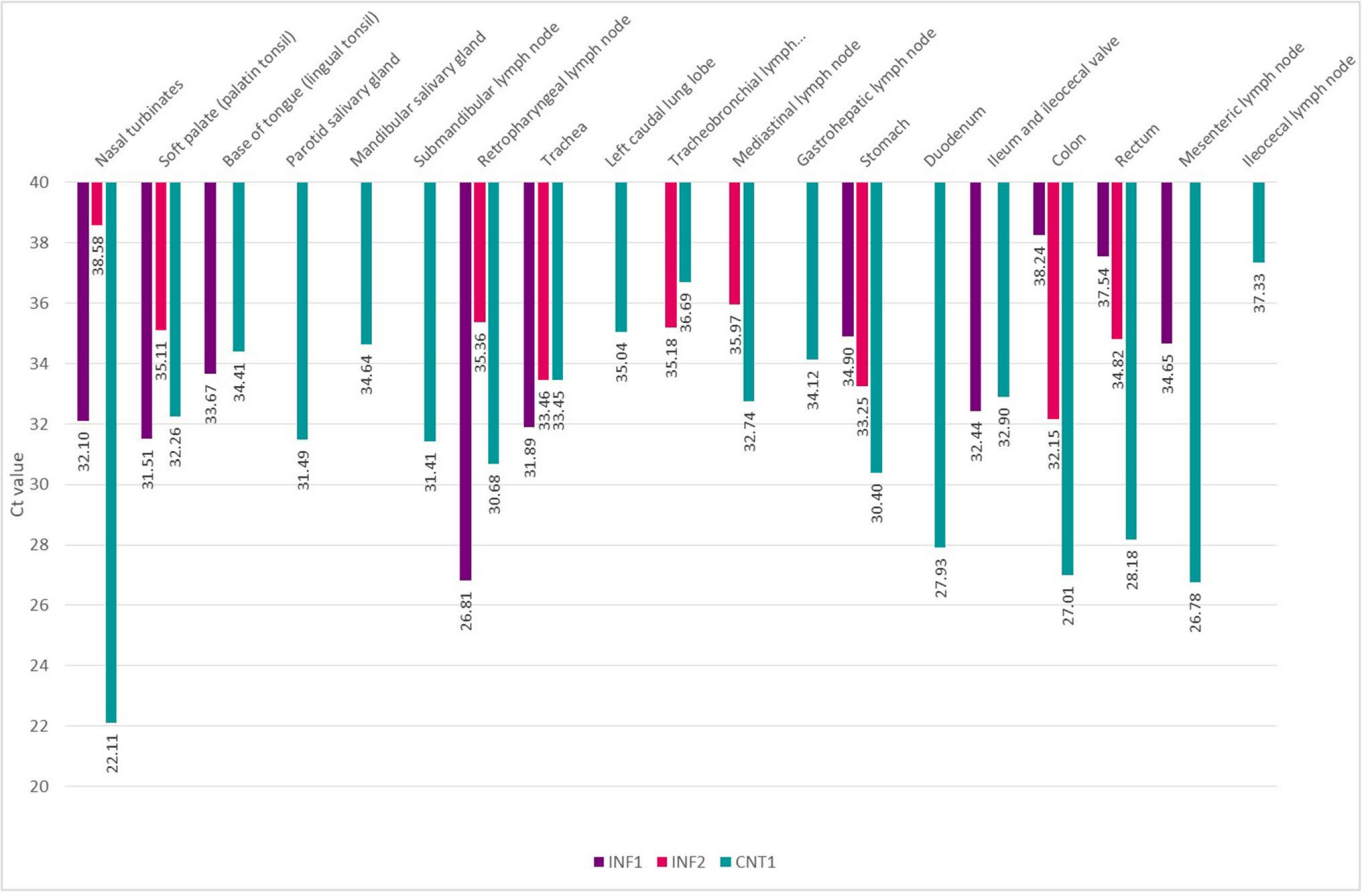
Viral loads based on average Ct values in tissues obtained from INF1 and INF2 on 11 DPI and CNT1 on 6 DPI*

Viral replication was detected in some of the tissues obtained from INF1 and INF2 (euthanized at 11 DPI) and in nearly all the tissues obtained from CNT1 (euthanized at 6 DPI). The lower Ct values (indicating higher viral loads) in INF1 were detected in the palatine and lingual tonsils, trachea, ileum and ileocecal valve, and retropharyngeal and mesenteric lymph nodes. In the case of INF2, the lower Ct values were detected in the trachea, stomach, colon, and rectum. Lower Ct values in CNT1 were detected in the nasal turbinates, parotid salivary gland, stomach, duodenum, colon, rectum, along with the submandibular, retropharyngeal, and mesenteric lymph nodes (Fig. 10). No viral RNA was detected in any of the tissues obtained from CNT2.

## Discussion

Many different experiments and scientific discussions have recently focused on SARS-CoV-2 infection in cats. Several cases of human-to-cat transmission have been reported throughout the world during the pandemic (Barroso-Arévalo et al. 2021; OIE 2021; Ruiz-Arrondo et al. 2020; Zhang et al. 2020). Although the number of cases is still low, this represents a risk in that cats may act as reservoirs for the disease, since the intermediate host involved in the virus jump to humans has not yet been identified. As cats are one of the most common pet species, it is, therefore, vital to discover their epidemiological role in the pandemic and their susceptibility under natural conditions. Furthermore, cats have been proposed as animal models for different studies on the virus, such as vaccine/treatment evaluations or in order to study the pathogenesis and epidemiology of the virus (Cleary et al. 2020; Takayama 2020). In this study, we focus on demonstrating that cats can become infected via routes of infection similar to those which occur under natural conditions, i.e., aerosol and oral transmission, by simulating sneezes/coughs and surface contamination. We also prove that transmission between infected and naïve cats does not occur if the air exchange is high, which should be taken into account when establishing preventive measures for pets.

In contrast with previous studies, we have attempted to replicate a natural infection, in which the cat is exposed to the virus by means of sneezes or coughs (aerosol), along with contaminated surfaces (oral infection). We, therefore, inoculated one cat (INF1) by spraying it with the virus (sneeze/cough infection model) and another cat (INF2) by distributing the virus over its hair (licking infection model). The dose used in this study (determined by TCID_50_ assay) was similar or even lower than that used in other experimental infections (Gaudreault et al. 2020; van den Brand et al. 2008). Both animals were successfully infected, and tested positive to PCR on oropharyngeal swabs from 1 DPI until the day of euthanasia, as has occurred in other studies in which animals were infected intranasally and orally (Gaudreault et al. 2020). No differences were found between the two routes of infection employed in terms of PCR results or gross lesions. The viral loads on oropharyngeal swabs, based on Ct values, achieved the highest values on 4DPI (INF1) and 6 DPI (INF2), while in other studies nasal swabs peaked on 3 DPI (Bosco-Lauth et al. 2020). The viral loads were stable and higher until 8 DPI. From 8 DPI to euthanasia (11DPI), the viral loads decreased in both animals, coinciding with the production of neutralizing antibodies, which is quite similar to that stated in previous reports in which antibody production started on 7 DPI (Bosco-Lauth et al. 2020; Gaudreault et al. 2020). Contrary to expectations, the animal infected by means of spraying (INF1) tested positive as regards the rectal swab on the three first days after infection, while the animal infected by the oral route (licking infection model, INF2) tested negative as regards the rectal swab in all the samplings. Keeping in mind that the route of infection in this last case was mainly digestive, it is surprising that the animal did not shed any virus in its feces. This suggests that viral replication is higher in the upper respiratory tract, regardless of the route of infection. In contrast with the results obtained in the case of rectal swabs, another study obtained positive results during all the experimental periods, starting on 3 DPI (Gaudreault et al. 2020), while yet another experiment that used a combination of inoculation routes (nasal, tracheal, oral and ocular) did not detect any viral RNA in feces (Halfmann et al. 2020).

However, the virus replicated in both the upper respiratory tract and the digestive mucosa, as shown by the PCR results obtained from tissues. Both INF1 and INF2, which underwent euthanasia on their respective 11 DPI, had viral RNA in their digestive tissues (stomach and rectum, among others) and in the upper respiratory tract (nasal turbinates, trachea, retropharyngeal lymph node), but not in the lower respiratory tract (lungs). These animals had severe vascular and cellular alterations in the lung, despite the fact that viral RNA was not detected in this organ. These pathological findings, such as congestion, edema, lymphoplasmacytic perivasculitis and vasculitis, increased numbers of type II pneumocytes, and alveolar macrophages. Alveolar septal thickening has also been observed in the lungs of ferrets when the virus is not present (Shi et al. 2020). A possible explanation for this might be that the lung lesions were triggered by an altered immune response, as has been reported in humans (Channappanavar and Perlman 2017; Mortaz et al. 2020). The current opinion regarding the pathogenicity of the virus is that SARS-CoV-2 induces the expression of numerous inflammatory factors, the maturation of dendritic cells, and the synthesis of type I interferons (IFNs). This aggravated response is responsible for pneumonia in humans and is characterized primarily by fever, cough, dyspnea, and bilateral infiltrates on chest imaging (Guan et al. 2020; Huang et al. 2020). Surprisingly, none of the infected animals in this study had any respiratory symptoms, despite the lesions observed in the nasal turbinates and lungs. These outcomes raise the question of whether the lesions would have recovered or evolved to a worse status in the following days. A recent study, in which several cats were euthanized 28 days post-infection, reported that moderate lesions persisted until that time, despite the clearance of the virus (Chiba et al. 2021). A response to this question could, therefore, be attained only by studying the progress of the lesions at different times in a long-term study. Some other lesions in the thymus, lymphoid nodes, and spleen were observed. These results coincided with those found in other studies, in which the splenic lymphoid follicles of the white pulp and the germinal centers of the lymph nodes of infected cats exhibited multifocal lymphocyte depletion (Bao et al. 2021; Chan et al. 2020). In the case of CNT1, which was infected via aerosol after not being infected in its sentinel contact period, a greater alveolar septal thickening associated with interstitial pneumonia was found, along with high viral shedding on oropharyngeal swabs, based on Ct values. This cat was euthanized on its 6 DPI*, and it might, therefore, be expected that the animal was in an acute phase of the infection. Viral RNA was detected in the majority of the tissues analyzed, suggesting that the virus was actively replicating. The viral RNA in this animal was detected in the lungs, although viral loads were low in the different lung lobes (targets’ average Ct value = 37.37). All these results are consistent with those of other studies in which the viral loads in the lungs were lower than those in the upper respiratory tract on 7 DPI (Gaudreault et al. 2020). However, in some studies, no viral RNA was detected in the lungs of the animals euthanized on their 6 DPI (Shi et al. 2020). As other studies have reported, the results of our study suggest that the infectious virus clears from the lower respiratory organs by day 10–11, but severe to moderate lesions persist in these tissues, despite virus clearance (Chiba et al. 2021).

With regard to symptomatology, none of the animals showed any specific symptoms, with the exception of INF2 (infected by oral route), which had diarrhea on 2 DPI, although, as described above, no viral RNA was detected on any of the rectal swabs taken from this animal. This absence of symptomatology is a common result in many other experiments (Bosco-Lauth et al. 2020; Halfmann et al. 2020; Martina et al. 2003; van den Brand et al. 2008). However, there is another study in which one animal between 70–100 days old died during the experiment on 3 DPI (Shi et al. 2020). This leads us to believe that younger cats are much more susceptible to SARS-CoV2 infection than juveniles or subadults. The temperature varied slightly over time, showing peaks at 4–5 DPI (INF1) and 2–3 DPI (INF2), but remained between the normal ranges most of the time. These outcomes are in line with those obtained in other studies, in which animals developed an asymptomatic infection with afebrile temperatures and recovered in about 10–11 days (Bosco-Lauth et al. 2020; Bosco-Lauth et al. 2020; Chiba et al. 2021; Gaudreault et al. 2020; Hobbs and Reid 2020).

In order to study potential transmissibility among cats under different air exchange conditions, we made three attempts at transmission in two different scenarios. The first involved the use of high air exchange. Two naïve cats were cohoused with two inoculated cats on 3 and 4 DPI, respectively (both inoculated animals were, according to the PCR results, shedding virus at that point). Neither of the two contact animals became infected, since all the samples taken were negative to PCR, except for the environmental sponges used to sample their hair. These environmental samples were positive in numerous samplings, which suggest that the virus was being shed from the infected animals. These results lead us to believe that viral release was occurring in sufficiently high doses to produce surface contamination but not to cause transmission between cats.

As the air exchange applied was high (scenario 1 = air renovation occurring 45 times per hour), another attempt was made with a lower air exchange (scenario 2 = air renovation occurring 22.5 times per hour). On this second attempt, we inoculated one cat (CNT1) and introduced another one the day after inoculation (CNT2). Again, the contact cat did not, according to the negative PCR results obtained for both the oropharyngeal and rectal swabs, blood, and after-necropsy tissues, become infected. This suggests that viral transmission may not occur among cats provided with high air exchange. These results contrast with those previously obtained in several studies, in which viral transmission was demonstrated among cats (Bosco-Lauth et al. 2020; Chiba et al. 2021; Halfmann et al. 2020; Hobbs and Reid 2020). However, none of the aforementioned studies provide details of air exchange conditions, and it is not, therefore, possible to compare our results with those attained in them. The air exchange used in this study was likely higher than the parameters used in other studies. This may explain why no aerosol transmission was detected during the experiment. Nevertheless, the animals were cohoused and, therefore, shared food and water. Despite being exposed to viral shedding, the sentinel cats did not become infected, thus showing that viral transmission between immunocompetent cats is not as frequent as might be expected.

Something that we can confirm is that, despite the fact that cats are susceptible to SARS-CoV-2 infection via natural routes of infection (aerosol and oral), viral transmission among cats does not seem to be likely providing that high air exchange is used. As pets normally live in environments with sufficient air renovation, their role as transmitters and reservoirs of the disease appears to be unclear. Taking into account the outcomes of our study, avoiding close contact with pets and maintaining good ventilation in COVID-19 positive houses may prevent viral transmissions from owners to their cats or transmission among cats. Furthermore, humans who are positive for SARS-CoV-2 virus should follow a very strict quarantine with their pets and not allow them to go outdoors in order to prevent them from coming into contact with other cats or wildlife and spreading the infection.

In conclusion, this paper provides new knowledge on COVID-19 infection in cats and viral transmission in this species. We have demonstrated that young cats are susceptible to viral infection by imitating natural routes of infection (sneezes, coughs, surface contamination) via aerosol and oral infection, since viral RNA was detected in several tissues, thus suggesting a systemic replication of the virus. However, the animals did not develop any clinical signs, with the exception of diarrhea in one cat, nor did they transmit the infection to other cats cohoused with them, taking into consideration the air exchange used (high air renovation). Surprisingly, despite finding moderate/severe lesions in the lungs, according to the after-necropsy PCR tests, the animals euthanized on 11 DPI did not show viral RNA in these organs. These lung lesions may, therefore, be a consequence of an aggravated immune system response. More studies on the progress of these lesions should be conducted in order to provide a response to these questions.

## Supplementary Information

Below is the link to the electronic supplementary material.

Supplementary file1 (JPG 51 KB) Additional file 1. Graphical representation of temperature of INF1 from its 0 day post-infection (DPI) until the day of euthanasia (11 DPI). Error bars indicate the 95% confidence interval.

Supplementary file2 (JPG 58 KB) Additional file 2. Graphical representation of temperature of INF2 from its 1 day post-infection (DPI) until the day of euthanasia (11 DPI). Error bars indicate the 95% confidence interval

Supplementary file3 (JPG 62 KB) Additional file 3. Graphical representation of temperature of CNT1 during its days as a sentinel contact for animal INF1 and its infection period until the day of euthanasia (6 day post-infection). Error bars indicate the 95% confidence interval

## Data Availability

The datasets used and/or analysed during the current study are available from the corresponding author on reasonable request.
