# Supplementary figures and images for: Evaluation of the clinical evolution and transmission of SARS-CoV-2 infection in cats by simulating natural routes of infection
Source: Vet Res Commun. 2022 Mar 3;46(3):837–52. doi: 10.1007/s11259-022-09908-5 (PMC8893356; doi:10.1007/s11259-022-09908-5)

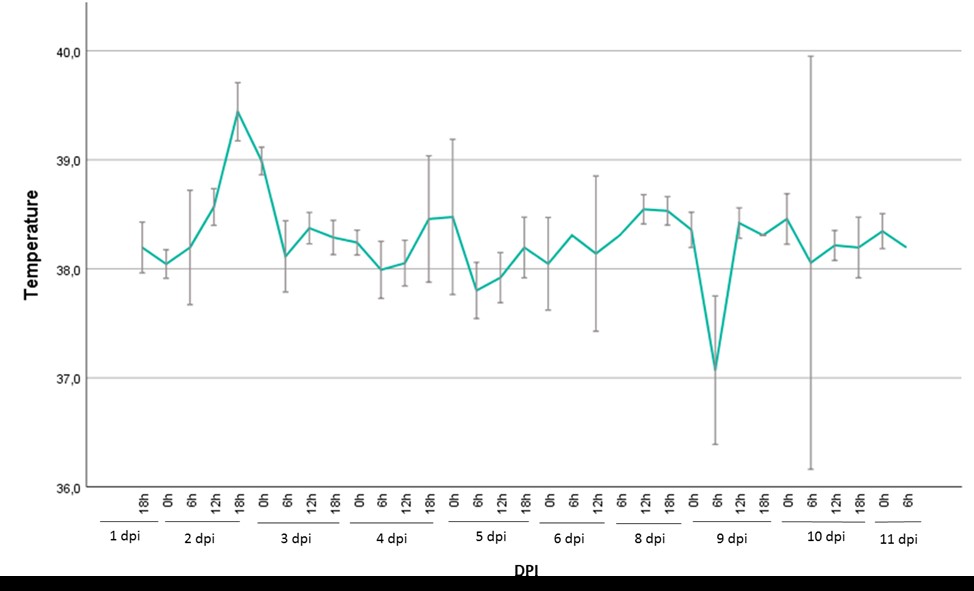

Supplement: Supplementary file 1 — Supplementary file1 (JPG 51 KB) Additional file 1. Graphical representation of temperature of INF1 from its 0 day post-infection (DPI) until the day of euthanasia (11 DPI). Error bars indicate the 95% confidence interval. [file 11259_2022_9908_MOESM1_ESM.jpg]

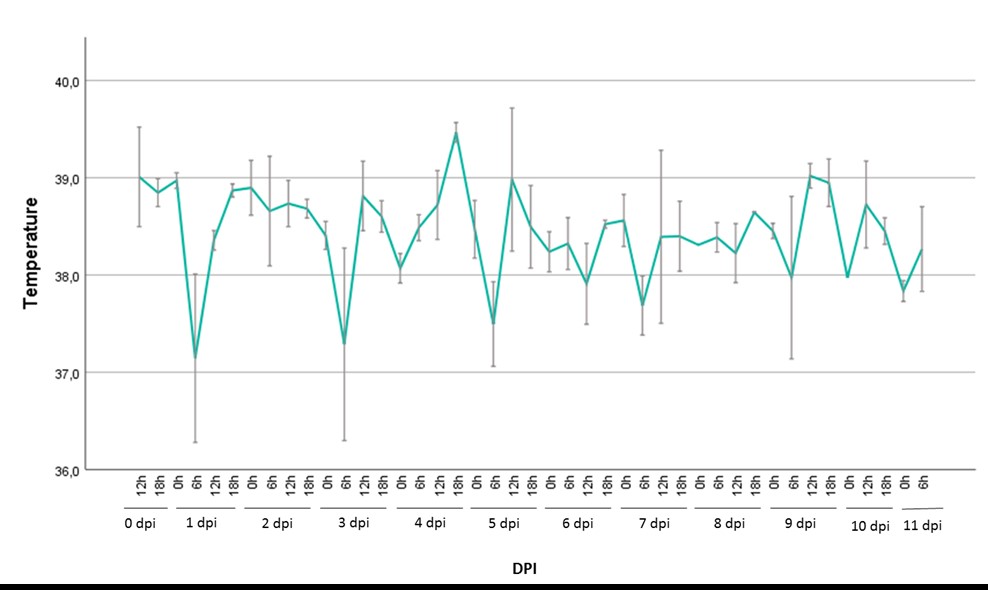

Supplement: Supplementary file 2 — Supplementary file2 (JPG 58 KB) Additional file 2. Graphical representation of temperature of INF2 from its 1 day post-infection (DPI) until the day of euthanasia (11 DPI). Error bars indicate the 95% confidence interval [file 11259_2022_9908_MOESM2_ESM.jpg]

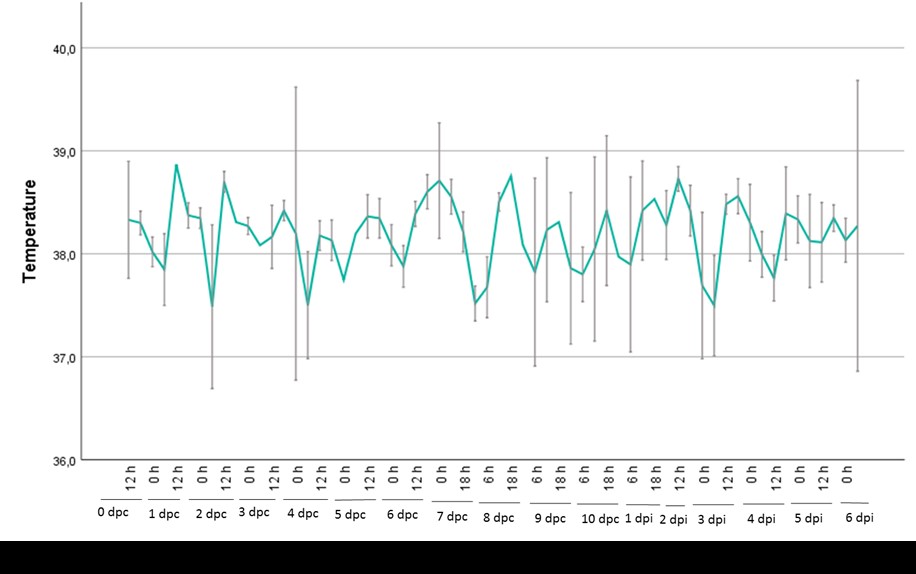

Supplement: Supplementary file 3 — Supplementary file3 (JPG 62 KB) Additional file 3. Graphical representation of temperature of CNT1 during its days as a sentinel contact for animal INF1 and its infection period until the day of euthanasia (6 day post-infection). Error bars indicate the 95% confidence interval [file 11259_2022_9908_MOESM3_ESM.jpg]
